# Overexpression of c-erbB2 is an independent marker of resistance to endocrine therapy in advanced breast cancer

**DOI:** 10.1038/sj.bjc.6690196

**Published:** 1999-03

**Authors:** S J Houston, T A Plunkett, D M Barnes, P Smith, R D Rubens, D W Miles

**Affiliations:** ICRF Clinical Oncology Unit, Guy's Hospital, London, SE1 9RT, UK

**Keywords:** c-erbB2, breast cancer, endocrine therapy, tamoxifen

## Abstract

The present study investigated the interaction between c-erbB2 overexpression and the response to first-line endocrine therapy in patients with advanced breast cancer. The primary tumours of 241 patients who were treated at first relapse with endocrine therapy were assessed for overexpression of c-erbB2 by immunohistochemistry. c-erbB2 was overexpressed in 76 (32%) of primary breast cancers and did not correlate with any other prognostic factor. The overall response to treatment and time to progression were significantly lower in patients with c-erbB2-positive tumours compared to those that were c-erbB2-negative (38% vs 56%, *P* = 0.02; and 4.1 months vs 8.7 months, *P* < 0.001, respectively). In multivariate analysis, c-erbB2 status was the most significant predictive factor for a short time to progression (*P* = 0.0009). In patients with ER-positive primary tumours treated at relapse with tamoxifen (*n* = 170), overexpression of c-erbB2 was associated with a significantly shorter time to progression (5.5 months vs 11.2 months, *P* < 0.001). In conclusion, overexpression of c-erbB2 in the primary tumour is an independent marker of relative resistance to first-line endocrine therapy in patients with advanced breast cancer. In patients with ER-positive primary tumours, the overexpression of c-erbB2 defines a subgroup less likely to respond to endocrine therapy. © 1999 Cancer Research Campaign


*British Journal of Cancer* (1999) **79**(7/8), 1220–1226
© 1999 Cancer Research Campaign
Article no. bjoc.1998.0196

### Overexpression of c-erbB2 is an independent marker of resistance to endocrine therapy in advanced breast cancer

**SJ Houston*, TA Plunkett*, DM Barnes, P Smith, RD Rubens and DW Miles**

ICRF Clinical Oncology Unit, Guy's Hospital, London SE1 9RT, UK

**Summary** The present study investigated the interaction between c-erbB2 overexpression and the response to first-line endocrine therapy in patients with advanced breast cancer. The primary tumours of 241 patients who were treated at first relapse with endocrine therapy were assessed for overexpression of c-erbB2 by immunohistochemistry. c-erbB2 was overexpressed in 76 (32%) of primary breast cancers and did not correlate with any other prognostic factor. The overall response to treatment and time to progression were significantly lower in patients with c-erbB2-positive tumours compared to those that were c-erbB2-negative (38% vs 56%, $P = 0.02$; and 4.1 months vs 8.7 months, $P < 0.001$, respectively). In multivariate analysis, c-erbB2 status was the most significant predictive factor for a short time to progression ($P = 0.0009$). In patients with ER-positive primary tumours treated at relapse with tamoxifen ($n = 170$), overexpression of c-erbB2 was associated with a significantly shorter time to progression (5.5 months vs 11.2 months, $P < 0.001$). In conclusion, overexpression of c-erbB2 in the primary tumour is an independent marker of relative resistance to first-line endocrine therapy in patients with advanced breast cancer. In patients with ER-positive primary tumours, the overexpression of c-erbB2 defines a subgroup less likely to respond to endocrine therapy.

**Keywords:** c-erbB2; breast cancer; endocrine therapy; tamoxifen

The c-erbB2 proto-oncogene encodes a 185 kD transmembrane glycoprotein and is a member of the epidermal growth factor (EGF) receptor family (Coussens et al, 1985). Amplification and/or overexpression of c-erbB2 has been reported in 10–40% of primary breast cancers, and has been associated with a worse prognosis in patients with node-positive (Slamon et al, 1987, 1989; Gullick et al, 1991) and node-negative disease (Gullick et al, 1991; Press et al, 1993, 1997; O'Malley et al, 1996; Charpin et al, 1997; Andrulis et al, 1998). A recent review proposed that its overexpression may be more predictive for overall survival than for disease-free survival (Clinical Practice Guidelines for the Use of Tumour Markers in Breast Cancer and Colorectal Cancer, 1996), suggesting that the prognostic effect of c-erbB2 overexpression may be on the response to anti-cancer treatment following relapse.

c-erbB2 may be a predictor of resistance to hormonal therapy in breast cancer. Several small studies demonstrated that c-erbB2 overexpression/amplification in patients with breast cancer was associated with a lower response rate and duration of response to first-line hormonal therapy at relapse (Wright et al, 1992; Klijn et al, 1993; Berns et al, 1995). However, other reports showed no statistically significant relationship between c-erbB2 overexpression and response to treatment, and concluded that it had no role in therapeutic decision-making in advanced breast cancer (Archer et al, 1995; Elledge et al, 1998).

We undertook a large retrospective study to investigate the relationship between c-erbB2 overexpression in the primary breast cancer and the response to first-line endocrine therapy for recurrent disease. Previous studies have demonstrated a correlation between c-erbB2 overexpression in the primary tumour and at metastatic sites (Niehans et al, 1993). The amplification and overexpression of the c-erbB2 proto-oncogene detected by Southern and Northern blotting have been shown to correlate with the c-erbB2 oncoprotein expression evaluated by Western blotting and immunohistochemistry (Slamon et al, 1987, 1989). Immunohistochemistry of the primary tumour is therefore an appropriate method for evaluating c-erbB2 gene dysregulation in this group of patients.

### MATERIALS AND METHODS

A total of 241 patients of the Breast Unit at Guy's Hospital who had received hormonal therapy as first-line treatment for recurrent breast cancer following primary surgery were identified from the database. The database contains information on patient and tumour characteristics, biological features such as histological grade and steroid receptor status, details of metastatic involvement, treatment and survival. The patients identified for this retrospective study had measurable or evaluable disease. Patients could have received prior adjuvant chemotherapy, but not prior adjuvant hormonal therapy or any previous treatment for metastatic disease.

Forty-three patients had received adjuvant chemotherapy. Sixteen had been treated with melphalan, while the others had received six cycles of cyclophosphamide (600 mg $m^{-2}$ intravenously (i.v.)), methotrexate (40 mg $m^{-2}$ i.v.) and 5-fluorouracil (600 mg $m^{-2}$ i.v.) on days 1 and 8 of each 28-day cycle (CMF).

Histological grading was performed using the criteria of Bloom and Richardson with modifications as suggested by Elston (1984). Oestrogen (ER) and progesterone receptor status were determined

*Received 21 April 1998*
*Accepted 3 August 1998*

*Correspondence to:* TA Plunkett

*Joint first authors.

**Table 1** Characteristics of patients with c-erbB2-negative or c-erbB2-positive primary tumours

| | c-erb-B2-negative (*n* = 165) | | c-erb-B2-positive (*n* = 76) | |
|---|---|---|---|---|
| Age | | | | |
| Median | 53 | | 54 | |
| Minimum | 21 | | 25 | |
| Maximum | 84 | | 76 | |
| | *n* | % | *n* | % |
| Menstrual status | | | | |
| Pre | 64 | 39 | 25 | 33 |
| Peri | 36 | 22 | 12 | 16 |
| Post | 63 | 38 | 36 | 47 |
| Uncertain | 3 | 1 | 2 | 3 |
| Pregnant/lactating | | | 1 | 1 |
| Tumour size | | | | |
| ≤ 2 cm | 32 | 20 | 17 | 24 |
| > 2 cm and ≤5 cm | 112 | 68 | 47 | 65 |
| > 5 cm | 20 | 12 | 8 | 11 |
| Unknown | 1 | | 4 | |
| Stage | | | | |
| Operable, node negative | 51 | 31 | 19 | 25 |
| Operable, node positive | 111 | 67 | 56 | 74 |
| Operable, node unknown | 3 | 2 | 1 | 1 |
| Histological type and grade | | | | |
| Ductal grade I | 2 | 1 | 1 | 1 |
| Ductal grade II | 82 | 50 | 32 | 42 |
| Ductal grade III | 51 | 31 | 29 | 38 |
| Lobular | 23 | 14 | 7 | 8 |
| Other | 7 | 4 | 8 | 11 |
| ER status | | | | |
| ≤ 10 fmole/mg | 33 | 20 | 19 | 25 |
| > 10 fmole/mg | 132 | 80 | 57 | 75 |
| PR status | | | | |
| ≤ 10 fmole/mg | 56 | 39 | 34 | 52 |
| > 10 fmole/mg | 89 | 61 | 32 | 49 |
| Unknown | 20 | | 10 | |
| Adjuvant treatment | | | | |
| None | 122 | 74 | 53 | 70 |
| Chemotherapy | 43 | 26 | 23 | 30 |

using the dextran-coated charcoal ligand binding assay (King et al, 1979) with a value of > 10 fmol $mg^{-1}$ cytosol protein taken as positive.

Expression of c-erbB2 oncoprotein was assessed in 5-μm sections of formalin-fixed, paraffin-embedded primary tumour tissue using a streptavidin–biotin immunohistochemical technique. The sections were dewaxed and placed in 0.1% hydrogen peroxide in methanol (0.01 M) phosphate buffered saline (PBS) at pH 7.2 (5:3) to block endogenous peroxidase activity. After washing in distilled water followed by PBS, the sections were incubated with fetal calf serum (FCS)/PBS (1:4) for 10 min (in order to reduce non-specific staining with primary antibody). Sections were then coated with 21 N polyclonal antibody at dilutions of 1/400 and 1/1000 in PBS and left at room temperature overnight. 21N is a rabbit polyclonal antibody raised to the predicted amino acid sequence from residues 1243–1255 of the open reading frame of c-erbB2 (Gullick et al, 1987). The following day, sections were washed in PBS for 10 min and incubated with secondary antiserum (biotinylated porcine anti-rabbit immunoglobulin (Daktopatts)) at 1/500 dilution in PBS containing 15% FCS and 3% human serum for 30 min and then rewashed in PBS. Treatment with avidin–biotin peroxidase complex (Daktopatts ABC complex) followed for 30 min. Peroxidase activity was demonstrated using diamino–benzidine solution (Sigma) and the nuclei were counterstained with haematoxylin.

**Table 2** Overall response rate following first line endocrine treatment

| | | |
|---|---|---|
| **By c-erbB2 status** | | |
| | CR/PR/SD | PD |
| c-erbB2-negative | 92/165 (56%) | 73/165 (44%) |
| c-erbB2-positive | 29/76 (38%) | 47/76 (62%) |
| | $\chi^2$ = 5.76, df = 1, *P* = 0.02 | |
| **By ER status** | | |
| | CR/PR/SD | PD |
| ER-negative | 10/52 (19%) | 42/52 (81%) |
| ER-positive | 111/189 (58%) | 78/189 (42%) |
| | $\chi^2$ = 23.9, df = 1, P < 0.0001 | |

Overall response rate was defined as complete response (CR), partial response (PR) or stable disease (SD) of more than 6 months duration. All other responses were classified as progressive disease (PD). df, ER, oestrogen receptor.

The specificity of the reaction was confirmed by abolition of staining following pre-incubation of antibody with the immunizing peptide. Negative controls in which PBS replaced the primary antiserum were run with each batch. A previously identified strongly staining tumour was used as a positive control. Inter- and intra-assay consistency was maintained by using these controls with each experiment. Any assay in which either control was unsatisfactory was repeated.

Previous work had demonstrated no prognostic differences between weak and strongly staining breast cancers (Barnes et al, 1988), thus tumours demonstrating any membrane staining were regarded as positive. Positive membrane staining was defined as coloured reaction product delineating the margins of tumour cells giving a 'fish-net' pattern (for original photograph see Barnes et al, 1988). All cases were reviewed by two pathologists and any disagreements were resolved by consultation. The pathologists were blinded to treatment outcome.

At relapse, systemic treatment for post-menopausal patients was tamoxifen 20 mg daily. Pre-menopausal patients were treated with ovarian irradiation, surgical oophorectomy or tamoxifen 20 mg daily. The predominant site of relapse was loco-regional soft tissue in 34%, skeletal in 26%, pleural/pulmonary in 21%, visceral in 5% and at other sites in 14% of patients.

Complete response (CR), partial response (PR) and stable disease (SD) were defined according to UICC criteria (Hayward et al, 1977). Overall response to treatment was defined as a patient having either CR, PR or SD of more than 6 months. The latter category was included as patients with stable disease have a similar survival to those showing a partial response (Manni et al, 1989). The time to progression was defined as the time from initiation of hormonal therapy to progression of disease, discontinuation of treatment or death.

Differences between categorical variables were determined using the $\chi^2$ test. The Yates correction was used for 2 × 2 tables. Time to progression was calculated according to the method of Kaplan and Meier. The resulting curves were compared using the log rank test. Multivariate analysis was performed using Cox's proportional hazards method.

© Cancer Research Campaign 1999

**Table 3** Overall response rate by ER and c-erbB2 status

| | ER-positive | ER-negative |
|---|---|---|
| c-erb-B2-positive | 15/57 (24%) | 1/19 (5%) |
| c-erb-B2-negative | 85/132 (64%) | 8/33 (24%) |

Overall response rate was defined as complete response (CR), partial response (PR) or stable disease of more than 6 months duration (SD). There is a significant difference in overall response rate between ER-positive–c-erbB2-positive and ER-positive–c-erbB2-negative cases ($P$ = 0.05). There is no difference between the ER-negative cases when divided by c-erbB2 status ($P$ = 0.29). ER, oestrogen receptor.

### RESULTS

The primary tumour from 76 women (32%) stained positively for c-erbB2. The relationships between c-erbB2 status and established prognostic features are shown in Table 1. There were no statistically significant differences between the two groups, in particular there was no statistically significant relationship between c-erbB2 status and tumour grade ($P$ = 0.32). There was a trend towards an inverse relationship between c-erbB2 overexpression and ER status ($P$ = 0.08) and between c-erbB2 overexpression and PR status ($P$ = 0.11).

Of the patients with c-erbB2-negative primary tumours, 139 (84%) received tamoxifen, three (2%) oophrectomy and 23 (14%) ovarian ablation at relapse. For the patients with c-erbB2-positive primary tumours, 72 (95%) were treated with tamoxifen and four (5%) with ovarian ablation at relapse.

The objective response rate (CR + PR) was significantly greater in patients with ER-positive or PR-positive tumours compared to those that were negative (ER 42% vs 17%, $P$ = 0.002; PR 43% vs 26%, $P$ = 0.01). The objective response rates in patients with c-erbB2-positive tumours were not different from those that were c-erbB2-negative (29% vs 40%, $P$ = 0.1).

When stable disease for greater than 6 months was included in the overall response category, the response to treatment was significantly greater in patients with c-erbB2-negative primary tumours (56 vs 38%, $P$ = 0.02), and in those that were ER-positive (58% vs 19%, $P$ < 0.0001; Table 2).

ER and c-erbB2 status were combined (Table 3). The response to endocrine treatment for patients with ER-positive–c-erbB2-positive primary tumours was significantly lower than that for ER-positive–c-erbB2-negative primary tumours (24% vs 64%, $P$ = 0.05). There was no difference in response rate when the ER-negative population was divided by c-erbB2 status (5% vs 24%, $P$ = 0.29).

The time to progression (TTP) was significantly shorter in patients with c-erbB2-positive tumours (median TTP 4.1 months), compared to those that were c-erbB2-negative (median TTP 8.7 months, $P$ < 0.001; Figure 1). The TTP was further analysed by univariate and multivariate Cox analyses. In both analyses c-erbB2-positive status was highly significantly associated with a shorter TTP (Table 4). The type of endocrine treatment had no effect on TTP in multivariate analysis ($P$ = 0.4).

A further analysis was performed to determine the influence of c-erbB2 status in patients with ER-positive tumours treated with tamoxifen. Of the 170 such patients, 54 (32%) were c-erbB2-positive. The TTP for ER-positive–c-erbB2-positive patients (median

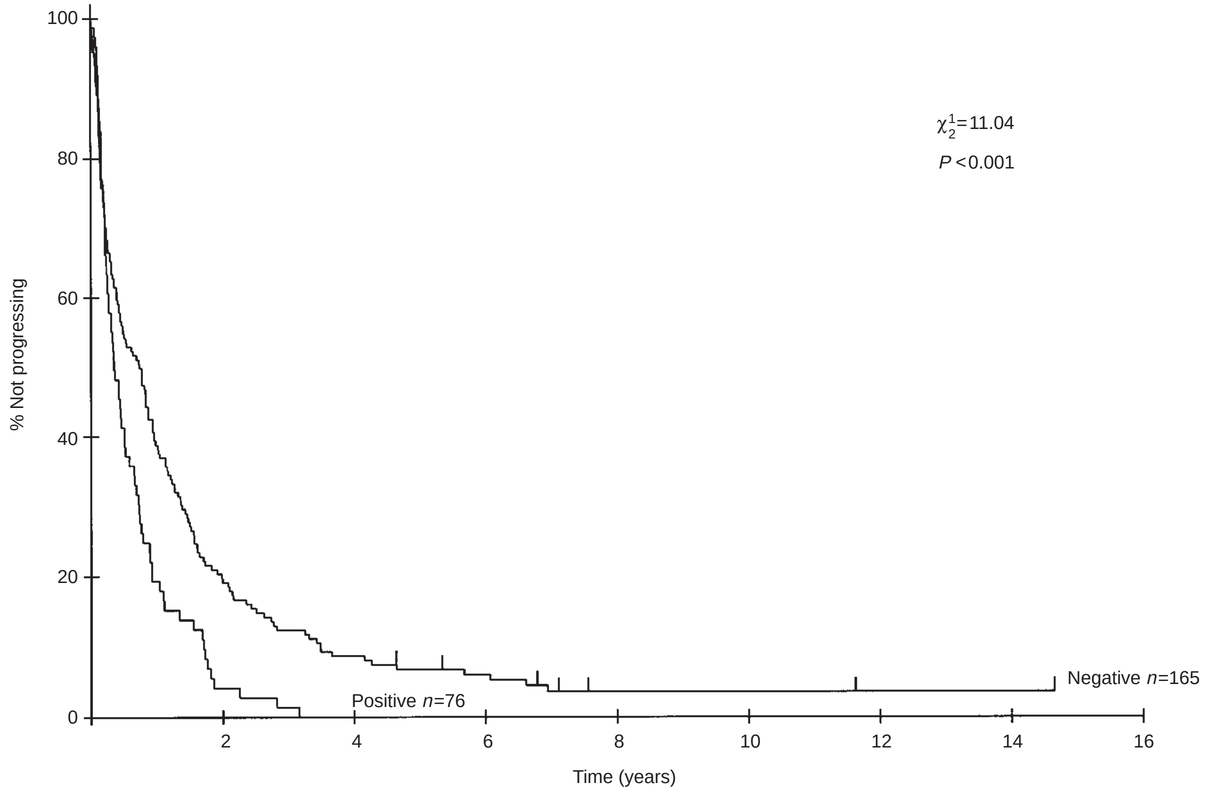

**Figure 1** Time to progression by c-erbB2 status

*© Cancer Research Campaign 1999*

**Table 4** Cox model analysis of time to progression

| Variable | Univariate $\chi^2$ | *P*-value | R.R.[a] | 95% C.I.[b] | Multivariate $\chi^2$ | *P*-value | R.R.[a] | 95% C.I.[b] |
|---|---|---|---|---|---|---|---|---|
| c-erbB2c | 11.40 | 0.007 | 1.69 | 1.26–2.26 | 11.08 | 0.009 | 1.69 | 1.25–2.29 |
| Age | 6.48 | 0.009 | 1.02 | 1.00–1.03 | 0.08 | 0.78 | 1.00 | 0.98–1.02 |
| Menstrual status[d] | 8.88 | 0.003 | 1.50 | 1.15–1.97 | 7.89 | 0.005 | 1.48 | 1.12–1.95 |
| Tumour size | 0.46 | 0.50 | 1.03 | 0.95–1.12 | 0.05 | 0.83 | 1.01 | 0.93–1.10 |
| Stage[e] | 0.44 | 0.51 | 1.10 | 0.83–1.47 | 0.39 | 0.53 | 1.11 | 0.80–1.53 |
| Histology[f] | 12.45 | 0.0004 | 1.65 | 1.25–2.16 | 9.42 | 0.002 | 1.54 | 1.17–2.03 |
| ER status[g] | 11.60 | 0.0007 | 1.82 | 1.32–2.52 | 7.85 | 0.005 | 1.64 | 1.18–2.29 |
| Adjuvant treatment[h] | 3.40 | 0.07 | 1.33 | 0.99–1.79 | 1.34 | 0.25 | 1.20 | 0.88–1.63 |

[a]Relative risk; [b]95% confidence intervals; [c]Negative v positive status; [d]Pre- vs post-menopausal; [e]Stage I v stage II disease; [f]Grade I v Grade II v Grade III; [g]Negative v positive status; [h]None v any

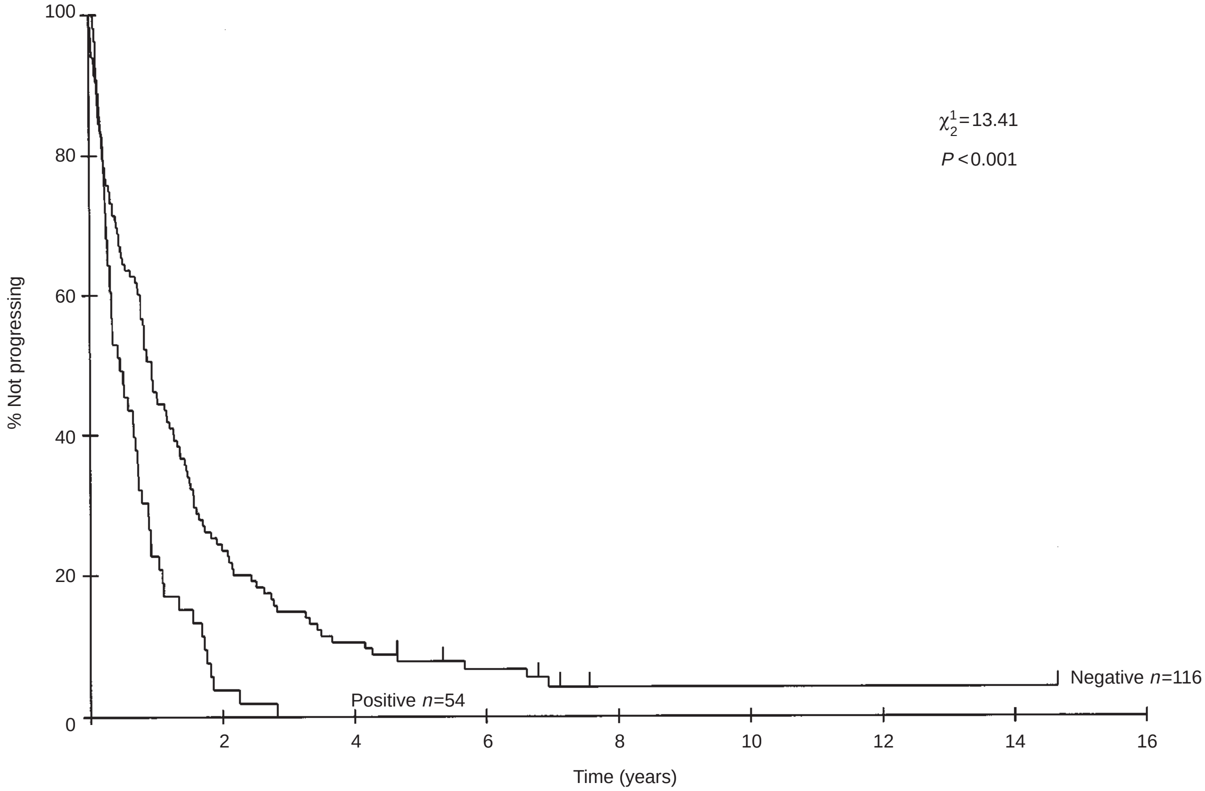

**Figure 2** Time to progression by c-erbB2 status. ER-positive patients treated with tamoxifen

TTP 5.5 months) was significantly shorter compared to that for ER-positive–c-erbB2-negative patients (median TTP 11.2 months), as shown in Figure 2 ($P < 0.001$). There was no difference in TTP when the ER-negative population was analysed according to c-erbB2 status (data not shown).

### DISCUSSION

In this study, positive membrane staining for c-erbB2 in the primary breast cancer predicted a low response rate and a short time to progression following first-line endocrine therapy for patients with recurrent disease. Positive c-erbB2 status was significantly associated with a shorter time to progression after adjusting for other known predictive factors such as menstrual status, oestrogen and progesterone receptor status, histological grade and site of relapse. Multivariate analysis demonstrated that c-erbB2 status was the most significant predictive factor for a short time to progression after initiation of endocrine therapy. In patients with ER-positive primary tumours, c-erbB2 overexpression defined a subgroup less likely to respond to endocrine therapy.

The c-erbB2 status of the primary tumour did not influence the objective response rate (CR + PR) to endocrine therapy. However, when stable disease of greater than 6 months duration was included, c-erbB2 overexpression was associated with a reduced

*© Cancer Research Campaign 1999*

overall response rate to treatment. Stable disease was included in the overall response assessment, as one-quarter of patients had predominantly skeletal disease. The response assessment is difficult in this group; the overexpression of c-erbB2 was still associated with a lower overall response rate after exclusion of patients with skeletal metastases (data not shown). Also, survival in patients with stable disease of greater than 6 months duration is similar to that of patients that achieve a PR to treatment (Manni et al, 1989). Others groups studying similar patients defined 'response' as CR + PR + SD of more than 6 months duration (Wright et al, 1992; Klijn et al, 1993; Archer et al, 1995; Berns et al, 1995; Elledge et al, 1998), and most demonstrated that overexpression of c-erbB2 reduced the response rate to endocrine therapy in advanced breast cancer.

As with all retrospective studies, there may have been unexpected bias. All patients had relapsed following primary surgery, had evaluable or assessable disease and received endocrine therapy. As ER expression in the primary tumour is a strong predictor of response to endocrine therapy in breast cancer (Rubens and Hayward, 1980), it is not surprising that most patients in this study had ER-positive primary tumours. ER expression has been reported to negatively correlate with c-erbB2 overexpression in an unselected population of patients with breast cancer (Ciocca et al, 1992). In the current study there was a trend towards ER and PR positivity with c-erbB2 negativity. Although the analysis was based on the tumour characteristics at presentation rather than at relapse, evidence suggests that c-erbB2 overexpression is similar in primary and metastatic disease (Niehans et al, 1993). Although ER loss has been reported, in most patients with ER-positive primary tumours the recurrent disease is also ER-positive (Kuukasjarvi et al, 1996).

Other workers have used immunohistochemistry to assess c-erbB2 status and have demonstrated resistance to endocrine therapy for patients with recurrent disease (Wright et al, 1992). In addition, several studies (Borg et al, 1994; Tetu and Brisson, 1994; Sjogren et al, 1998), and two randomized trials (Carlomagno et al, 1996; Stal et al, 1997), have demonstrated that c-erbB2 overexpression in the primary breast cancer reduced the benefit from adjuvant tamoxifen.

Two studies reported that c-erbB2 overexpression did not alter response to hormonal therapy (Archer et al, 1995; Elledge et al, 1998). The former was based on fewer than 100 patients and so lacked statistical power. The study of Elledge et al used 205 ER-positive patients from the Southwest Oncology Group (SWOG) study 8228 (Ravdin et al, 1992). Their study population differs from ours in several respects. Their criterion for ER positivity was set at the lower level of > 3 fmol $mg^{-1}$ cytosol; in practice, at our and other institutions, the clinical cut-off value for ER-positivity in the ligand binding assay was usually > 10 fmol $mg^{-1}$ (Barnes et al, 1998). All patients in the study of Elledge et al received tamoxifen as initial therapy for metastatic disease. However, 47% of patients had metastatic disease at presentation (Elledge et al, 1998) whereas all patients in our study had relapsed following surgery. In the SWOG study c-erbB2 status was defined by a scoring system for membrane staining of tumour cells. Any tumour with more than 1 in 100 tumour cells stained was deemed c-erbB2-positive. In the study that we report, any membrane staining was regarded as positive for c-erbB2.

In their initial analysis c-erbB2 overexpression did not influence either response rate or time to progression (Elledge et al, 1998). However, because of issues of reproducibility in the immunohistochemistry, a second independent observer scored the slides. Using the same scoring systems, in the repeat analysis c-erbB2 overexpression significantly reduced the time to progression (median TTP 4 months vs 8 months, $P = 0.01$). This highlights the problems of scoring systems and inter-observer variation in immunohistochemical assessment of pathological specimens, and undermines their conclusion that c-erbB2 overexpression is not associated with a poorer response to tamoxifen (Elledge et al, 1998). In the study we report, any membrane staining was regarded as positive and all slides were examined by two pathologists (who were blinded to the response data); in patients with ER-positive tumours treated with tamoxifen, c-erbB2 overexpression significantly reduced TTP (5.5 months vs 11.2 months, $P < 0.001$).

Other investigators have used serum erbB2 estimates in breast cancer patients. The extracellular domain of the c-erbB2 protein has been found in the sera of 20–40% of patients with metastatic breast cancer (Leitzel et al, 1992; Kath et al, 1993). In separate studies using different assays for serum erbB2, elevated pre-treatment levels predicted a poor response to first-line (Yamauchi et al, 1997) and second-line endocrine therapy in patients with metastatic breast cancer (Leitzel et al, 1995). These studies did not determine the relationship between serum levels of erbB2 and tissue expression.

Experimental evidence suggests that c-erbB2 overexpression may modulate response to endocrine therapy. Studies in vitro and in animal models using the MCF-7 human breast cancer cell line have demonstrated that transfection with c-erbB2 reduced their sensitivity to tamoxifen (Benz et al, 1992; Pietras et al, 1995). In experimental studies, overexpression and/or stimulation of c-erbB2 down-regulated ER expression and activity (Pietras et al, 1995; Saceda et al, 1996), but other mechanisms have been proposed.

The function of c-erbB2 may not be to bind any ligand directly, but rather to increase the binding affinity of ligands to heterodimers of the erb family (Karunagaran et al, 1996). c-erbB2 can form heterodimers with the EGF receptor (erbB1) and neu differentiation factor (NDF) receptors (erbB3 and erbB4). The overexpression of c-erbB2 in cell lines reduced the ligand dissociation rate for both EGF and NDF, whereas the removal of c-erbB2 accelerated the dissociation rate. The prolonged binding of EGF or NDF resulted in increased activation of the measured cytoplasmic kinases. This suggests that c-erbB2 may affect the response to a wide variety of ligands by altering the binding affinities of growth factor receptors, and its overexpression may result in a growth advantage for affected cells.

The oestrogenic stimulation of breast cancer cells involves growth factors for the erb family of receptors (Kern et al, 1990). It has been demonstrated that patients who had either EGF receptor-positive and/or c-erbB2-positive disease had a significantly lower response rate to endocrine therapy than those who were negative for both (Newby et al, 1997). The overexpression of c-erbB2 may result in some oestrogenic growth factor pathways becoming independent of the ER, and explain our observations of relative resistance to endocrine therapy in such cases.

If c-erbB2 overexpression is a marker of resistance to endocrine therapy in breast cancer, it may be that these women would be better treated with chemotherapy. There are conflicting data on whether overexpression of c-erbB2 predicts for resistance to different combinations of chemotherapy. Several studies have suggested that c-erbB2 overexpression may be a marker for resistance to treatment with adjuvant CMF (Allred et al, 1992; Gusterson et al, 1992). However, both of these studies included an endocrine component in the adjuvant therapy. In the study of

 © Cancer Research Campaign 1999

Gusterson et al, low-dose prednisolone plus tamoxifen was added to chemotherapy, and in the study of Allred et al, prednisolone was added to chemotherapy. The lack of efficacy of adjuvant treatment in the c-erbB2-positive groups may have been due to differences in the response to the endocrine component of the adjuvant therapy. Others have reported that c-erbB2 overexpression predicted for a better response to CMF chemotherapy in the treatment of metastatic breast cancer (Klijn et al, 1993; Berns et al, 1995).

The current study has shown that c-erbB2 overexpression, determined by immunohistochemistry in the primary tumour, predicted for relative resistance to endocrine therapy in patients with recurrent breast cancer. Other studies have demonstrated that overexpression of c-erbB2 negates the benefits of adjuvant tamoxifen in patients with ER-positive primary tumours (Carlomagno et al, 1996; Stal et al, 1997). These observations may influence the choice of systemic therapy for breast cancer in patients whose primary tumour overexpressed c-erbB2. A logical extension would be to inhibit both the ER and c-erbB2 pathways. Experimental studies using the BT474 human breast cancer line (which expresses ER and overexpresses c-erbB2) demonstrated that the combination of tamoxifen and 4D5 (a monoclonal antibody to c-erbB2) inhibited cell growth significantly more than either agent alone (Witters et al, 1997). The administration of antibody to c-erbB2 has proven effective in clinical trials of patients with primary breast cancers that overexpress c-erbB2 (Baselga et al, 1996). The combination of endocrine therapy and antibody to c-erbB2 may be worthy of investigation in patients with ER-positive tumours that also overexpress c-erbB2.

### REFERENCES

Allred DC, Clark G, Tandon A, Schnitt SJ, Gilchrist KW, Osborne CK, Tormey DC and McGuire WL (1992) HER-2/neu in node-negative breast cancer: prognostic significance of overexpression influenced by the presence of in situ carcinoma. *J Clin Oncol* **10**: 599–605

Andrulis IL, Bull SB, Blackstein ME, Sutherland D, Mak C, Sidlofsky S, Pritzker KP, Hartwick RW, Hinna W, Lickley L, Wilkinson R, Qizlbash A, Ambus U, Lipa M, Weizel H, Katz A, Baida M, Mariz S, Stoik G, Dacamara P, Strongitharm D, Geddie W and McCready D (1998) neu/erbB2 amplification identifies a poor-prognosis group of women with node-negative breast cancer. *J Clin Oncol* **16**: 1340–1349

Archer SG, Eliopoulos A, Spandidos D, Barnes D, Ellis IO, Blamey RW, Nicholson RI and Robertson JF (1995) Expression of ras p21, p53 and c-erbB2 in advanced breast cancer and response to first line hormonal therapy. *Br J Cancer* **72**: 1259–1266

Barnes DM, Lammie GA, Millis RR, Gullick WJ, Allen DS and Altman DG (1988) An immunohistochemical evaluation of c-erbB2 expression in human breast carcinoma. *Br J Cancer* **58**: 448–452

Barnes DM, Millis RR, Beex LVAM, Thorpe SM and Leake RE (1998) Increased use of immunohistochemistry for oestrogen receptor measurement in mammary carcinoma: the need for quality assurance. *Eur J Cancer* **34**: 1677–1682

Baselga J, Tripathy D, Mendelsohn J, Baugham S, Benz CC, Dantis L, Sklarin NJ, Seidman AD, Hndis CA, Moore J, Rosen PP, Twaddell T, Henderson IC and Norton L (1996) Phase II study of weekly intravenous recombinant humanized anti-p185HER2 monoclonal antibody in patients with HER2/neu-overexpressing metastatic breast cancer. *J Clin Oncol* **14**: 737–744

Benz CC, Scott GK and Sarup JC (1992) Estrogen-dependent, tamoxifen-resistant tumorigenic growth of MCF-7 cells transfected with HER2/neu. *Breast Cancer Res Treat* **24**: 85–95

Berns EMJJ, Foekens JA, van Staveren IL, van Putten WLJ, de Koning HYWCM, Portengen H and Klijn JGM (1995) Oncogene amplification and prognosis in breast cancer: relationship with systemic treatment. *Gene* **159**: 11–18

Borg A, Baldetorp B, Ferno M, Killander D, Olsson H, Ryden S and Sigurdsson H (1994) ERBB2 amplification is associated with tamoxifen resistance in steroid-receptor positive breast cancer. *Cancer Lett* **81**: 137–144

Carlomagno C, Perrone F, Gallo C, De Laurentis MD, Lauria R, Morabito A, Pettinato G, Panico L D'Antonio A, Bianco R and De Placido S (1996) c-erbB2 overexpression decreases the benefit of adjuvant tamoxifen in early-stage breast cancer without axillary lymph node metastases. *J Clin Oncol* **14**: 2702–2708

Charpin C, Garcia S, Bouvier C, Martini F, Lavaut M-N, Allasia C, Bonnier P and Andrac L (1997) c-erbB2 oncoprotein detected by automated quantitative immunocytochemistry in breast carcinomas correlates with patients' overall and disease-free survival. *Br J Cancer* **75**: 1667–1673

Ciocca DR, Fujimura FK, Tandon AK, Clark GM, Mark C, Lee-Chen GJ, Pounds GW, Vendely P, Owens MA, Pandian MR and McGuire WL (1992) Correlation of HER-2/neu amplification with expression and with other prognostic factors in 1103 breast cancers. *J Natl Cancer Inst* **84**: 1279–1282

Clinical Practice Guidelines for the Use of Tumor Markers in Breast and Colorectal Cancer (1996). *J Clin Oncol* **14**: 2843–2877

Coussens L, Yang-Feng TL, Liao TL, Chen E, Gray A, McGrath J, Seeburg PH, Libermann TA, Schlessinger J, Francke U, Levinson A and Ullrich A (1985) Tyrosine kinase receptor with extensive homology to EGF receptor shares chromosomal location with neu oncogene. *Science* **230**: 1132–1139

Elledge RM, Green S, Ciocca D, Pugh R, Allred CA, Clark GM, Hill J, Ravdin P, O'Sullivan J, Martino S and Osborne CK (1998) HER-2 expression and response to tamoxifen in estrogen receptor-positive breast cancer: a Southwest Oncology Group study. *Clin Cancer Res* **4**: 7–12

Elston CW (1984) The assessment of histological differentiation in breast cancer. *Aust NZ J Surg* **54**: 11–15

Gullick WJ, Love SB, Wright C, Barnes DM, Gusterson B, Harris AL and Altman DG (1991) c-erbB2 protein overexpression in breast cancer is a risk factor in patients with involved and uninvolved lymph nodes. *Br J Cancer* **63**: 434–438

Gusterson BA, Gelber RD, Goldhirsch A, Price KN, Save-Soderborgh J, Anbazhagan R, Styles J, Rudenstam C-M, Goloum R, Reed R, Martinez-Tello F, Tiltman A, Torhorst J, Grigolato P, Bettelheim R, Neville AM, Burki K, Castiglione M, Collins J, Lintner J and Senn H-J (1992) Prognostic importance of c-erbB2 expression in breast cancer. *J Clin Oncol* **10**: 1049–1056

Hayward JL, Carbone PP, Heuson JC, Kumaoka S, Segaloff A and Rubens RD (1977) Assessment of response to therapy in advanced breast cancer. *Cancer* **39**: 1289–1294

Karunagaran D, Tzahar E, Beerli RR, Chen X, Graus-Porta D, Ratzkin BJ, Seger R, Hynes NE and Yarden Y (1996) ErbB-2 is a common auxiliary subunit of NDF and EGF receptors: implications for breast cancer. *EMBO J* **15**: 254–264

Kath R, Hoffken K and Otte C (1993) The neu oncogene product in serum and tissue of patients with breast carcinoma. *Ann Oncol* **4**: 585–590

Kern FG, Cheville AL and Liu Y (1990) Growth factor receptors and the progression of breast cancer. *Semin Cancer Biol* **1**: 317–328

King RJB, Redgraves J and Hayward JL (1979) The measurement of receptors for oestradiol and proesterone in human breast tumours. In *Steroid Receptor Assays in Breast Tumours: Methodological and Clinical Aspects*, King RJB (ed), p. 55–73. Alpha-Omega: Cardiff

Klijn JGM, Berns EMJJ, Bontebal M and Foekens J (1993) Cell biological factors associated with the response of breast cancer to systemic treatment. *Cancer Treat Rev* **19SB**: 45–63

Kuukasjarvi T, Kononen J, Helin H, Holli K and Isola J (1996) Loss of estrogen receptor in recurrent breast cancer is associated with poor response to endocrine therapy. *J Clin Oncol* **14**: 2584–2589

Leitzel K, Teramoto Y, Sampson E, Mauceri J, Langton BC, Demers L, Podczaski E, Harvey H, Shambaugh S, Volas G, Weaver S and Lipton A (1992) Elevated soluble c-erbB2 antigen levels in the serum and effusions of a proportion of breast cancer patients. *J Clin Oncol* **10**: 1436–1443

Leitzel K, Teramoto Y, Konrad K, Chinchilli VM, Volas G, Grossberg H, Harvey H, Demers L and Lipton A (1995) Elevated serum c-erbB2 antigen levels and decreased response to hormone therapy of breast cancer. *J Clin Oncol* **13**: 1129–1135

Manni T, Trujillo JE and Marshall (1989) Anti-hormone treatment of stage IV breast cancer. *Cancer* **43**: 444–450

Newby JC, Johnston SRD, Smith IE and Dowsett M (1997) Expression of epidermal growth factor receptor and c-erbB2 during the development of tamoxifen resistance in human breast cancer. *Clin Cancer Res* **3**: 1643–1651

Niehans GA, Singleton TP and Dykoski D (1993) Stability of HER-2/neu expression over time and at multiple metastatic sites. *J Natl Cancer Inst* **5**: 1230–1235

O'Malley FP, Saad Z, Kerkvliet N, Doig G, Stitt L, Ainsworth P, Hundal H, Chambers AF, Turnbull DI and Bramwell V (1996) The predictive power of semiquantitative immunohistochemical assessment of p53 and c-erbB2 in lymph node negative breast cancer. *Hum Pathol* **27**: 955–963

Pietras RJ, Arboleda J and Reese DM (1995) HER-2 tyrosine kinase pathway targets estrogen receptor and promotes hormone-independent growth in human breast cancer cells. *Oncogene* **10**: 2435–2446

© Cancer Research Campaign 1999

Press MF, Pike MC, Chazin VR, Hung G, Udove JA, Markowicz M, Danyluk J, Godolphin W, Sliwkowski M, Akita R, Brandeis J, Paterson MC and Slamon DJ (1993) Her-2/neu expression in node-negative breast cancer: direct tissue quantitation by computerized image analysis and association of overexpression with increased risk of recurrent disease. *Cancer Res* **53**: 4960–4970

Press MF, Bernstein L, Thomas PA, Meisner LF, Zhou J-Y, Ma Y, Hung G, Robinson RA, Harris C, El-Naggar A, Slamon DJ, Philips RN, Ross JS, Wolman SR and Flom KJ (1997) HER-2/neu gene amplification characterised by fluoresence in situ hybridisation: poor prognosis in node-negative breast carcinomas. *J Clin Oncol* **15**: 2894–2904

Ravdin PM, Green S, Door TM, McGuire WL, Fabian C, Pugh RP, Carter RD, Rivkin SE, Borst JR, Belt RJ, Metch B and Osborne CK (1992) Prognostic significance of progesterone receptor levels in estrogen receptor-positive patients with metastatic breast cancer treated with tamoxifen: results of a prospective SWOG study. *J Clin Oncol* **10**: 1284–1291

Rubens RD and Hayward JL (1980) Estrogen receptors and response to endocrine therapy and cytotoxic chemotherapy in advanced breast cancer. *Cancer* **46**: 2922–2924

Saceda M, Grunt TW, Colomer R, Lippman ME, Lupu R and Martin MB (1996) Regulation of estrogen receptor concentration and activity by an erbB/HER ligand in breast carcinoma cell lines. *Endocrinology* **137**: 4322–4330

Sjogren S, Inganas M, Lindgren A, Holmberg L and Bergh J (1998) Prognostic and predictive value of c-erbB2 overexpression in primary breast cancer, alone and in combination with other prognostic markers. *J Clin Oncol* **16**: 462–469

Slamon DJ, Clark GM, Wong SG, Levin WJ, Ullrich A and McGuire WL (1987) Human breast cancer: correlation of relapse and survival with amplification of the HER-2/neu oncogene. *Science* **235**: 177–182

Slamon DJ, Godolphin W, Jones LA, Holt JA, Wong SG, Keith DE, Levin WJ, Stuart SG, Udove J, Ullrich A and Press MF (1989) Studies of the HER-2/neu proto-oncogene in human breast cancer and ovarian cancer. *Science* **244**: 707–712

Stal O, Ferno M, Borg A and Nordenskjold B (1997) ERBB2 expression and benefit from 5 versus 2 years of adjuvant tamoxifen for postmenopausal stage II breast cancer patients. *Breast Cancer Res Treat* **46**: 32 (abstr)

Tetu B and Brisson J (1994) Prognostic significance of the pattern of immunostaining and adjuvant therapy. *Cancer* **73**: 2358–2365

Witters LM, Kumar R, Chinchilli VM and Lipton A (1997) Enhanced anti-proliferative activity of the combination of tamoxifen plus HER-2-neu antibody. *Breast Cancer Res Treat* **42**: 1–5

Wright C, Nicholson S, Angus B, Sainsbury JRC, Farndon J, Cairns J, Harris AL and Horne C (1992) Relationship between c-erbB2 protein product expression and response to endocrine therapy in advanced breast cancer. *Br J Cancer* **65**: 118–121

Yamauchi H, O'Neill A, Gelman R, Carney W, Tenney DY, Hosch S and Hayes DF (1997) Prediction of response to antiestrogen therapy in advanced breast cancer patients by pretreatment circulating levels of extracellular domain of the HER-2/c-neu protein. *J Clin Oncol* **15**: 2518–2525

*© Cancer Research Campaign 1999*
